# Clone-Specific Variation in *Myzus persicae* Influences Transmission of BMYV and BYV and Associated Feeding Behavior

**DOI:** 10.3390/insects16080784

**Published:** 2025-07-30

**Authors:** Grégoire Noël, Lallie Glacet, Christiane Then, Frédéric Francis

**Affiliations:** Laboratory of Functional and Evolutionary Entomology, University of Liège, Gembloux Agro-Bio Tech, 5030 Gembloux, Belgium; lallie.glacet@uliege.be (L.G.); frederic.francis@uliege.be (F.F.)

**Keywords:** ELISA, electropenetrography, aphid–plant interaction, *Beta vulgaris*, feeding behavior

## Abstract

Sugar beet (*Beta vulgaris* ssp. *vulgaris*) is a vital crop, contributing to nearly a quarter of global sugar production, but faces significant challenges from biotic stressors, particularly aphids, which transmit damaging yellowing viruses such as Beet Yellow Virus (BYV) and Beet Mild Yellowing Virus (BMYV). Following the partial ban of neonicotinoids in Europe, viral infections in sugar beet have surged, highlighting the need for a deeper understanding of aphid-mediated virus transmission mechanisms. This study aims to evaluate the transmission efficiency of BYV and BMYV through different clones of the aphid vector *Myzus persicae* from sugar beet seed companies across Europe, and to analyze the feeding behaviors of efficient clones to identify factors influencing virus transmission. The transmission rates of yellowing viruses by *M. persicae* clones ranged from 52% to 79% for BMYV (mean 65%) and 7% to 96% for BYV (mean 47%). While no significant differences in BMYV transmission efficiency were observed among clones, a significant difference was detected between two BYV-carrying clones. Moreover, the BYV-carrying clone exhibited prolonged penetration activities during its feeding phase compared to the BMYV-carrying clone, suggesting a potential behavioral influence on transmission efficiency. This study highlights the importance of considering aphid clone influence in the development of sugar beet resistance.

## Simple Summary

Sugar beet is a crucial crop in worldwide agricultural production. However, its productivity is increasingly threatened by piercing-sucking insects, particularly aphids, which transmit yellowing viruses. The partial ban of neonicotinoids in Europe has exacerbated viral infections, underscoring the urgent need to understand aphid-mediated virus transmission mechanisms. This study investigated the transmission efficiency of Beet Yellow Virus (BYV) and Beet Mild Yellowing Virus (BMYV) by various clones of *Myzus persicae* sourced from different sugar beet seed companies across Europe. Contrasted transmission rates were observed among aphid clones, with different prolonged penetration behaviors identified as a key factor. These findings underscore the importance of aphid clone behavior in virus transmission and emphasize the need to incorporate clone-specific dynamics into sugar beet resistance strategies. By informing targeted breeding programs, this research contributes to enhancing crop resilience and ensuring sustainable production of sugar beet.

## 1. Introduction

Cultivated across approximately fifty countries, sugar beet (*Beta vulgaris* ssp. *vulgaris* Linnaeus 1753) is a crop of significant economic value, primarily utilized for sugar production and, to a lesser extent, bioethanol. Contributing to 20–25% of global sugar output, this vital crop is subjected to numerous biotic threats, including nematodes, leafhoppers, and particularly aphids [1,2]. Aphids (Hemiptera: Aphididae) are of particular concern due to their role as vectors of viral diseases such as yellowing viruses, which can severely reduce crop yields [3]. Until recently, extensive use of neonicotinoids provided effective aphid control in beet cultivation. However, following their partial prohibition by the European Union in 2018 [4], an increase in virus infections has been reported by growers (personal communication).

The yellowing virus complex comprises several distinct virus species, including Beet Yellow Virus (BYV), Beet Mild Yellowing Virus (BMYV), Beet chlorosis Virus (BchV), and Beet Mosaic Virus (BtMV) [5,6,7]. Beet Yellow Virus (BYV) or *Closterovirus flavibetae* is a semi-persistent virus characterized by a single-stranded RNA genome and a filamentous morphology, approximately 1300 nm in length. It is a member of the genus Closterovirus within the family Closteroviridae [8]. BYV is associated with severe yellowing symptoms, and upon infection, it accumulates in the phloem tissue, significantly disrupting nutrient transport. This disruption manifests as reduced photosynthetic capacity, leading to stunted plant growth [8]. In contrast, Beet Mild Yellowing Virus (BMYV), also known as *Polerovirus BMYV*, belongs to the family Luteoviridae and the genus Polerovirus. BMYV is transmitted persistently and typically induces milder yellowing symptoms compared to BYV [9]. Unlike BYV, which is a non-circulative virus, BMYV is classified as a circulative virus. Despite these differences, both viruses are phloem-limited, residing primarily within the phloem cells of infected plants.

In Europe, these virus pathogens are mainly transmitted by certain aphid species, notably *Myzus persicae* (Sulzer, 1776), also known as the green peach aphid [2,5,7,10,11,12]. The transmission efficiency of aphid vectors, including *M. persicae*, depends on their ability to effectively reach host plants, inoculate phytoviruses, and develop large aphid populations throughout the cultivation season [13]. Early infestations in sugar beet crops are particularly dangerous, potentially leading to severe viral epidemics, especially in mild winter and warm spring conditions. The green peach aphid exhibits high efficiency in transmitting BYV, BMYV, and BChV, with rates ranging from 28 to 100% for Poleroviruses and 51 to 73% for BYV [5]. Apterous individuals show higher transmission rates of BYV (78.57%) compared to winged forms (57.15%) [14]. It is well known that the efficiency of the viral transmission is dependent on the aphid species and tested virus strain [15]. However, on a lower taxonomic approach, little scientific information assessed the transmission efficiency of plant viruses according to different aphid clones over cultivated plant species except for wheat [16] and cucumber [17], and nothing is known about the impact of aphid clones on the transmission of yellowing viruses for sugar beet plants.

The evaluation of the optimum inoculation access period and acquisition access period for the vectorization of BMYV and BYV by *M. persicae* remains challenging [18,19], with limited studies addressing these parameters [14]. Existing studies suggest that the optimal timing is influenced by aphid density per experimental plant, which affects virus acquisition levels over varying durations [20,21]. Differences in the stylet activities of *M. persicae* that are associated with successful inoculation or acquisition of sugar beet yellowing viruses may explain these variations. Additionally, factors such as retention sites within the vector, transmission strategies, and the feeding behaviors of aphids likely play a significant role in determining transmission rates. The electropenetrography (EPG) technique has been widely employed to identify specific stylet penetration (probing) activities in aphids related to virus transmission [22]. By monitoring aphid feeding behavior through EPG, researchers can deduce the stylet tip positions within plant tissues and identify specific stylet activities through the analysis of distinct electrical waveform patterns from aphid behavior [14,23].

The objectives of this study are to assess the transmission efficiency of two sugar beet yellowing viruses (i.e., BMYV and BYV) and several clones of *M. persicae* originating from different European sugar beet seed companies. After selecting efficient aphid clones for the transmission of BMYV and BYV, the feeding behaviors of these clones were compared regarding the different sugar beet yellowing viruses.

## 2. Materials and Methods

This work is part of a research project on the development of control methods for sugar beet yellowing viruses in collaboration with IRBAB (Institut Royal Belge pour l’Amélioration de la Betterave). All aphid rearings took place in the entomology laboratories of the Gembloux Agro-Bio Tech faculty (ULiège, Belgium).

### 2.1. Susceptible Beta vulgaris L. Var. Saccharifera Cultivation

All the seeds were supplied by IRBAB (Tienen, Belgium), which in turn purchased them from various seed companies. Only plant varieties considered as susceptible to sugar beet yellowing virus transmission were used in this study.

The sugar beet seedlings were sown in 3 cm square plastic pots in “La Plaine Chassart” (50 L, Wagnelée, Fleurus, Belgium) potting soil. After 1 or 2 weeks, the plants were transplanted into 5 cm × 5 cm × 5 cm square pots. The various phases of plant growth were carried out in Bugdom© nylon cages (MegaView Science Co., Ltd., Taichung, Taiwan) measuring 92 cm × 47.5 cm × 47.5 cm. Above each cage was a lighting system consisting of LEDs with 16 h of light per day (1.9 × 1.1 × 101 cm, 23 W, Vegeled Bi-phosphorous white 4 K-N1 Spectrum, 2950 Lm/m, 128LED 2835/m, 24VDC CV, 1 m cbl), one meter long, connected by transformers (Meanwell encl. switch. Pow. sup. IP67 Full AC-DC in active PFC 320 W 24 VDC tun, Guangzhou, China). The temperature was 21 ± 2 °C with 70% relative humidity. Temperature and humidity were maintained throughout the experiment.

### 2.2. Myzus Persicae Rearing

The aphids originate from different laboratories and correspond to several distinct clones, each named according to their origin (Table 1). In total, eight aphid clones were tested for their potential to transmit BMYV and BYV. Aphids were reared on plants whose infection with BMYV or BYV was confirmed by an ELISA test with an absorbance value greater than 0.300. Rearing took place at a temperature of 22 ± 2 °C, and the cages used to contain the aphids were Bugdom© nylon cages measuring 92 × 47.5 × 47.5 cm, similar to those used for sugar beet. The aphids were subjected to a 16 h photoperiod at 70% RH. To avoid contamination among aphid clones, each clone was maintained on separate, individually caged host plants within the growth chamber. The cages were physically isolated to prevent any accidental transfer between colonies. Handling tools were disinfected between uses and dedicated to specific clones during transfers. In addition, the personnel followed strict clone-specific handling protocols to minimize cross-contact. No evidence of contamination was observed throughout the experimental period, and colony identities were routinely checked based on known performance and morphological consistency.

### 2.3. Transmission Tests

The transmission test was conducted using five adult aphid clones reared under the conditions described above. Adult aphids belonging to the same clone were moved from the source plant to the test plant using a brush. This operation was carried out on a set of 15 plants of the same variety. A complete experiment comprises three sets of 15 plants of the same variety, each carrying five aphids that had previously acquired either BYV or BMYV. The aphids were left on the plant for 48 h under aphid rearing conditions. The plants were then sprayed with Multisect, an acetamide-based insecticide (a systemic insecticide from the neonicotinoid family) from the “KB” brand (Evergreen Garden Care, Lokeren, Belgium). Once the aphids were eliminated, the plants were placed in an incubation chamber for three weeks. In this chamber, the temperature was 22 ± 1 °C and the relative humidity was 70 ± 10%. A double-antibody sandwich (DAS)-ELISA was performed to detect virus infection in beet plants used in the transmission tests. For virus detection, we used ELISA kits from LOEWE Biochemica GmbH (Sauerlach, Germany). BYV was detected using kit reference 07012, which includes specific polyclonal antibodies for both capture and detection, diluted 1:250 in the coating and conjugate buffers. For BMYV, we used kit reference 07009, which targets Turnip yellows virus (TuYV) and detects BMYV and Beet western yellows virus (BWYV) due to their strong antigenic similarity. This kit also contains two essential antibodies (capture and detection), used at the same 1:250 dilution.

Leaf tissue samples (0.5 g from four disks per plant) were ground in extraction buffer and 0.2 mL of homogenate was added to each well. Plates were incubated at 4 °C for 16 h, washed, and then incubated with alkaline phosphatase-conjugated secondary antibodies diluted 1:250 for 4 h at 37 °C.

Substrate solution (p-nitrophenyl phosphate) was added, and after color development for 4 h under light, optical density was measured at 405 nm using a microplate reader (Bio-Rad, Hercules, CA, USA). Samples with OD_405_ values above 0.299 were considered positive.

### 2.4. DC Electropenetrography

DC electropenetrography (EPG) is an electrophysiological technique used to characterize the feeding behavior of piercing-sucking insects by monitoring their stylet penetration activities within plant tissues. The method relies on differences in electrical potential between plant tissues (e.g., xylem, phloem, mesophyll) to distinguish distinct feeding phases. The copper nails were connected to the headstage amplifier of a Giga-8d DC-EPG system (EPG Systems, Wageningen, The Netherlands), operating at an input resistance (Ri) of 10^9^ Ω with an applied DC signal typically ranging between 50 and 100 mV [23,24]. The EPG system consists of a closed electrical circuit formed by a plant electrode inserted into the potting substrate, a gold wire (6 cm length, 25 μm diameter) connecting the aphid to a signal amplifier via a copper nail (5 cm length), and the tested wingless aphid at adult stage, affixed to the gold wire using an adhesive mixture composed of silver powder (5–8 μm, 99.9% purity; Sigma-Aldrich, Darmstadt, Germany), “Tesa glue pen” adhesive, and distilled water (1:1:1 ratio).

Plants (*n* = 22) were individually enclosed in Faraday cages to mitigate electromagnetic interference (Figure S1). Prior to the experiment, aphids were starved for 1 h. Each aphid, attached to the EPG system via the gold wire, was then placed on a sugar beet plant, allowing limited but natural movement necessary for probing and feeding behavior. The experimental setup included inverted pots with Petri dish lids filled with water to maintain hydration. Electrical signals were continuously recorded for 8 h using the EPG Stylet+d software version 1.2 (EPG system, Wageningen University, Wageningen, The Netherlands). Waveform analysis was conducted using the A2EPG software version 5.4.0 [25]. We examined aphid probing behavior on the study plants by analyzing the following EPG parameters: non-penetration (NP), cell punctures by the stylet (pd), the stylet pathway phase (a combination of waveforms A, B, and C), mechanical activity and difficulties during stylet penetration (waveform F), xylem ingestion (waveform G), and the phloem phase (waveform E). The phloem phase includes two sub-phases: salivation into the sieve elements (E1) and phloem sap ingestion (E2). This experiment was designed to compare feeding behavior from selected *M. persicae* clones combined with one of the beet yellowing viruses: Myz_1-BMYV and Myz_K-BYV.

### 2.5. Statistical Analysis

Due to the unbalanced experimental design (Table 2), Generalized Linear Mixed Models (GLMMs) were employed to compare the transmission rates of the two yellowing viruses across all selected *M. persicae* clones [26]. The ELISA 96-well plates and plant batches were included as random effects to account for potential variability. A binomial error distribution was used to model the transmission outcome, defined as 1 for an infected sugar beet plant and 0 for a non-infected plant after exposure to infected aphids. For pairwise comparisons among aphid clones, estimated least-squares means were calculated for BMYV and BYV using the *emmeans* package in R version 4.3.1 [27]. To compare the transmission rates of BMYV and BYV for individual aphid clone, additional GLMMs were fitted using the same random effect structure. In this analysis, the transmission event was modeled according to virus type, followed by an estimated least-squares means test.

To evaluate differences in aphid probing behavior among species, we analyzed the proportion of time spent in specific EPG waveforms, including pathway phase (C), derailed stylet mechanics (F), xylem ingestion (G), salivation in sieve elements (E1), and phloem ingestion (E2). For each parameter, linear mixed-effects models (LMMs) were fitted using the lmer function from the *lme4* package in R [28], with aphid species as a fixed effect and recording session as a random effect. Model assumptions were checked using residual diagnostics. We assessed the significance of fixed effects using Type II ANOVA. Post hoc pairwise comparisons between aphid species were performed using estimated marginal means (EMMs), adjusted for multiple testing with Tukey’s HSD method, and implemented via the *emmeans* package [27]. Compact letter displays (CLDs) were generated to visualize significant group differences using the *multcomp* and *emmeans* packages [29].

## 3. Results

**Figure 1:**
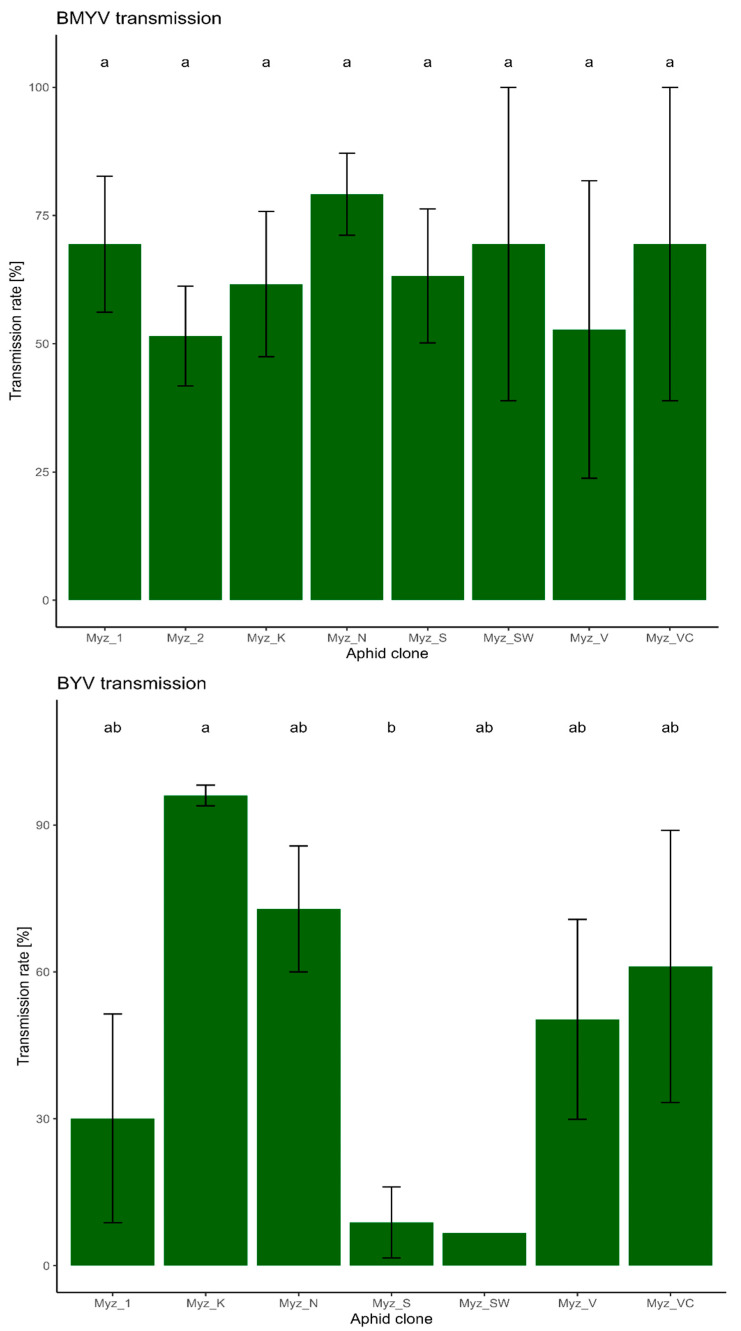
Barplot of transmission rate [%] ± SE per aphid clone for BMYV (**above**) and BYV (**below**). Different minor letters indicate significant differences (*p* < 0.05) among the aphid clones for each virus transmission, as determined by the estimated marginal means test using a Generalized Linear Mixed Model with a binomial error distribution.

A total of 837 plant samples were analyzed for virus transmission using ELISA (Table 2). The observed transmission rates ranged from 52% to 79% for BMYV, with a mean of 65%, and from 7% to 96% for BYV, with a mean of 47% (Table 3). No significant differences in transmission efficiency were detected among the aphid clones for BMYV. However, one significant difference in transmission efficiency (z ratio = 3.43; *p*-value = 0.011) was observed between the aphid clones Myz_K and Myz_S for BYV (Figure 1). Additionally, for each aphid clone, the transmission rates were consistent between BMYV and BYV (Table 3).

**Figure 2:**
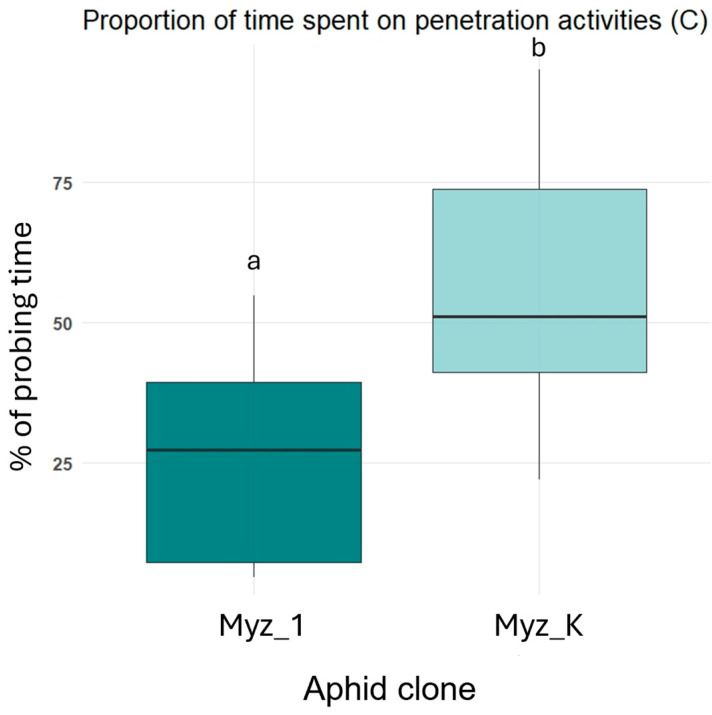
Percentage of time spent on penetration activities (C waveform) by two *Myzus persicae* clones (Myz_1 and Myz_K) as measured by electrical penetration graph (EPG) recordings. Different letters indicate statistically significant differences between clones (linear mixed-effects model followed by Tukey’s HSD test, *p* < 0.05).

**Figure 3:**
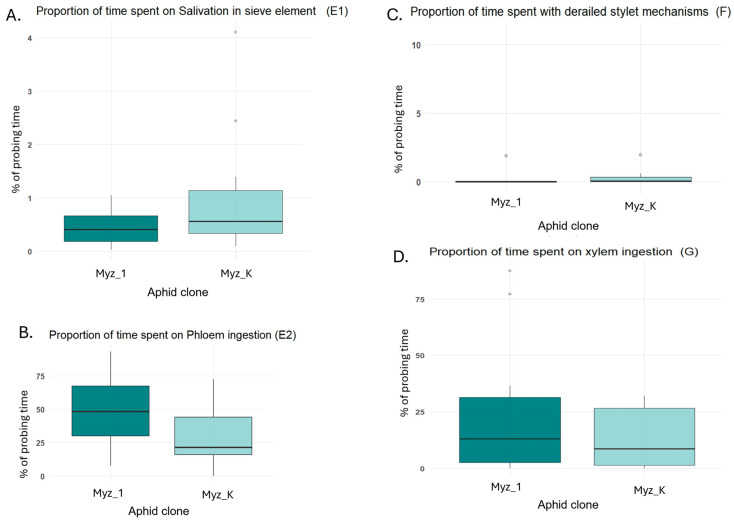
Percentage of time spent in distinct feeding phases by two *Myzus persicae* clones (Myz_1 and Myz_K) as measured by electrical penetration graph (EPG) recordings. (**A**) Salivation into sieve elements (E1), (**B**) phloem ingestion (E2), (**C**) derailed stylet mechanics (F), and (**D**) xylem ingestion (G).

Clone Myz_K spent a significantly greater proportion of probing time engaged in penetration activities compared to Myz_1 (F = 13.06; *p*-value < 0.05). Estimated marginal means (±SE) were 55.5 ± 6.1% for Myz_K and 23.1 ± 6.7% for Myz_1, with no overlap in group letters (a ≠ b), indicating a statistically significant difference (Figure 2). No significant difference was observed in salivation behavior between clones (F = 2.18; *p*-value > 0.05). Myz_K showed a mean salivation time of 1.04 ± 0.28%, while Myz_1 showed 0.42 ± 0.31%, both in the same statistical group (Figure 3A). Although Myz_1 tended to spend more time ingesting phloem (49.6 ± 9.0%) than Myz_K (29.3 ± 8.2%), this difference was not statistically significant (F = 2.94; *p*-value > 0.05), and both clones were assigned the same group label (Figure 3B). There was no significant difference between clones in the proportion of time spent in derailed stylet mechanics (F = 0.83; *p*-value > 0.05). Both clones were assigned to the same group (a), with mean percentages of 0.29 ± 1.2% (Myz_K) and 1.85 ± 1.4% (Myz_1) (Figure 3C). The proportion of time spent on xylem ingestion did not differ significantly between clones (F = 1.40; *p*-value > 0.05). Mean values were 14.0 ± 7.3% for Myz_K and 26.5 ± 7.9% for Myz_1, with overlapping confidence intervals and shared group assignments (Figure 3D).

## 4. Discussion

BMYV and BYV are significant viruses that pose serious threats to sugar beet cultivation in Europe, with *M. persicae* being one of the most prominent vectors of phytoviruses [5]. This study focused on both virus species and the diversity of aphid clones originating from different seed companies. Various factors have previously been identified as influencing virus transmission, including virus strains, aphid species, vector dynamics, life-history traits, and the source and host plant species from which the aphids were collected or maintained [13,30,31].

Transmission efficiency of BMYV was relatively consistent among aphid clones, aligning with values reported in previous studies [10,11]. In contrast, BYV showed greater variability in transmission across clones and generally lower transmission rates than previously published results [10,14]. Such variability underscores the importance of aphid clone identity in shaping transmission outcomes, consistent with earlier findings highlighting intra-species variation in vector competence [16,17,32].

A key behavioral difference was observed between the BYV-transmitting clone Myz_K and BMYV-transmitting Myz_1. Myz_K exhibited a significantly longer duration of penetration-related activity (C waveform), suggesting more extensive stylet navigation through non-phloem tissues. This contrasts with established trends for non-circulative virus vectors, which typically show shorter pathway phases to optimize quick inoculation [33]. BYV is a semi-persistent virus, while BMYV is circulative, and these differences in transmission mode are known to shape vector behavior [34]. However, our results suggest that clone-specific factors may override expected virus-related patterns, particularly in early feeding stages.

Such behavioral divergence aligns with prior studies on *Acyrthosiphon pisum* (Harris, 1776), where genetically distinct clones displayed different probing and phloem access behaviors—even when feeding on identical host plants [35]. Similarly, research on *Rhopalosiphum padi* (L.) and *Sitobion avenae* (Fabricius, 1775) has shown that transmission efficiency and EPG parameters vary significantly across clones, even under controlled conditions [36,37]. In *S. avenae*, proteomic differences between high- and low-efficiency clones were associated with transmission ability, although only a few proteins out of thousands were differentially expressed [38]. Despite its widespread role as a polyphagous virus vector, no comparable clone-level behavioral or proteomic studies have yet been conducted on *M. persicae*. This highlights a notable gap in our understanding of intra-species variability in this important vector, and the need for more detailed investigations.

Additionally, physiological status such as starvation stress could also explain longer pathway phases observed in clone Myz_K. Starved aphids have been shown to allocate more time to water regulation and exhibit less efficient feeding patterns [39]. This effect may be amplified or modulated by clone-specific traits, suggesting an interaction between environmental stress and inherent behavioral tendencies.

Interestingly, no significant differences were observed in the phloem-related EPG phases (E1, E2, F, G) between the two virus-carrying clones. This suggests that once phloem access was achieved, both clones showed similar salivation and ingestion behavior—indicative of equivalent phloem acceptability and feeding efficiency. Although Myz_1 (BMYV) showed a non-significant trend toward longer E2 (phloem ingestion), this should be interpreted cautiously. As emphasized by Walker (2024) [40], such trends may result from biological variation or experimental noise but could still point to subtle differences in feeding strategy, phloem composition, or virus-induced modifications.

This study reinforces the need to account for aphid clone-specific behaviors when interpreting virus–vector dynamics. Clone-level variability complicates predictions based solely on virus transmission mode and highlights the necessity of using well-characterized vector genotypes in virus transmission studies. Furthermore, the observed differences underscore the potential for selecting efficient vector clones in experimental setups aimed at understanding sugar beet resistance and virus spread.

## Figures and Tables

**Table 1:** Aphid clone details. The country of origin is given for sugar beet seed companies.

Myzus persicae Clone	Seeder Clone Origin
Myz_N	Netherlands
Myz_S	Germany
Myz_K	Germany
Myz_V	Belgium
Myz_1	Belgium
Myz_2	Belgium
Myz_SW	Denmark
Myz_VC	Belgium and France

**Table 2:** Number of ELISA replicates for virus transmission tests combining *M. persicae* clone and yellowing viruses.

Clone	BMYV	BYV
Myz_N	92	29
Myz_S	97	64
Myz_K	108	58
Myz_V	50	79
Myz_1	58	59
Myz_2	42	0
Myz_SW	27	16
Myz_VC	28	30
**Total**	**502**	**335**

**Table 3:** Mean transmission rate [%] (± SE) among aphid clones for BMYV and BYV. Different minor letters indicate significant differences (*p* < 0.05) among the aphid clones for each virus transmission, as determined by the estimated marginal means test using a Generalized Linear Mixed Model with a binomial error distribution. NA indicates that the SE was not applicable.

Aphid clone	BMYV	BYV
Myz_N	79.16 ± 8.01 a	72.86 ± 12.86 ab
Myz_S	63.23 ± 13.05 a	8.83 ± 7.28 b
Myz_K	61.63 ± 14.16 a	96.03 ± 2.1 a
Myz_V	52.78 ± 29 a	50.29 ± 20.42 ab
Myz_1	69.42 ± 13.26 a	30.08 ± 21.32 ab
Myz_2	51.52 ± 9.74	-
Myz_SW	69.44 ± 30.56 a	6.67 ± NA ab
Myz_VC	69.44 ± 30.56 a	61.11 ± 27.78 ab

## Data Availability

To preserve the anonymity of the seed manufacturers that provide the aphid clones, data and analyses are available upon request from the paper’s authors.

## Supplementary Materials

The following supporting information can be downloaded at: https://www.mdpi.com/article/10.3390/insects16080784/s1, Figure S1: EPG set-up and electrode to the aphid dorsum.
