# Supplementary figures and images for: Clone-Specific Variation in Myzus persicae Influences Transmission of BMYV and BYV and Associated Feeding Behavior
Source: Insects. 2025 Jul 30;16(8):784. doi: 10.3390/insects16080784 (PMC12386179; doi:10.3390/insects16080784)

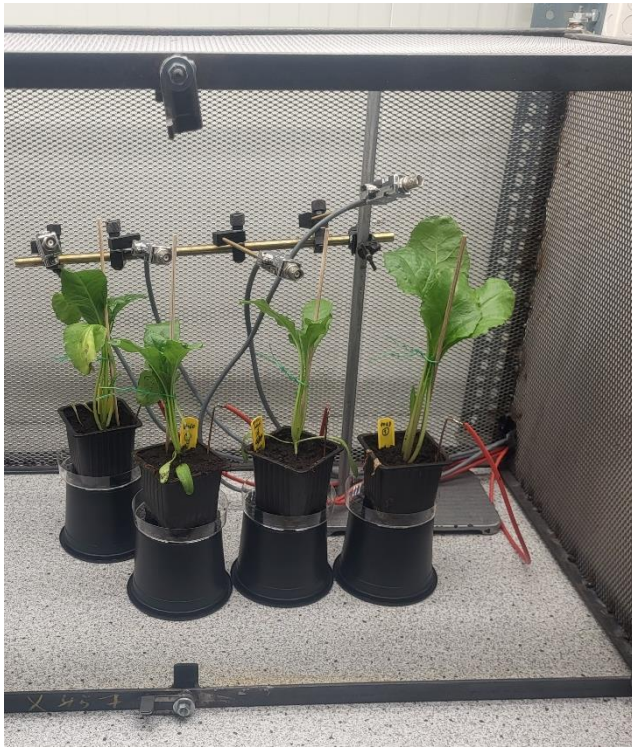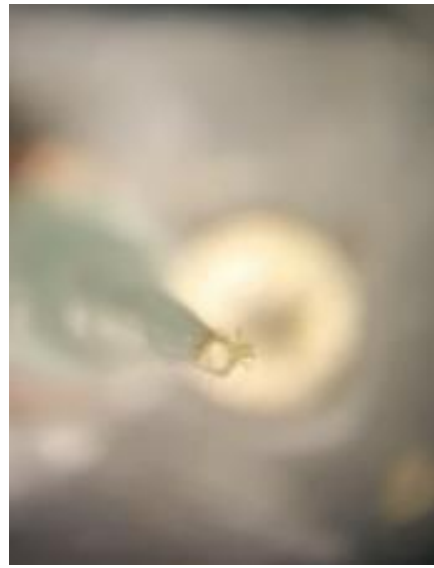

Figure S1 : EPG set-up (left) and electrode to the aphid dorsum (right). Credit photo: Lallie Glacet.

Supplement: Supplementary file 1 [file insects-16-00784-s001.zip › insects-3672685-supplementary.pdf]
